# Crystal Structure Confirmation of JHP933 as a Nucleotidyltransferase Superfamily Protein from *Helicobacter pylori* Strain J99

**DOI:** 10.1371/journal.pone.0104609

**Published:** 2014-08-07

**Authors:** Yanhe Zhao, Xianren Ye, Yintao Su, Lifang Sun, Feifei She, Yunkun Wu

**Affiliations:** 1 State Key Laboratory of Structural Chemistry, Fujian Institute of Research on the Structure of Matter, Chinese Academy of Sciences, Fuzhou, Fujian, China; 2 Key Laboratory of Ministry of Education for Gastrointestinal Cancer, School of Basic Medical Sciences, Fujian Medical University, Fuzhou, Fujian, China; Veterans Affairs Medical Center (111D), United States of America

## Abstract

*Helicobacter pylori* is a well-known pathogen involved in the development of peptic ulcer, gastric adenocarcinoma and other forms of gastric cancer. Recently, there has been more considerable interest in strain-specific genes located in plasticity regions with great genetic variability. However, little is known about many of these genes. Studies suggested that certain genes in this region may play key roles in the pathogenesis of *H. pylori*-associated gastroduodenal diseases. JHP933, a conserved putative protein of unknown function, is encoded by the gene in plasticity region of *H. pylori* strain J99. Here we have determined the structure of JHP933. Our work demonstrates that JHP933 is a nucleotidyltransferase superfamily protein with a characteristic αβαβαβα topology. A superposition demonstrates overall structural homology of the JHP933 N-terminal fragment with lincosamide antibiotic adenylyltransferase LinA and identifies a possible substrate-binding cleft of JHP933. Furthermore, through structural comparison with LinA and LinB, we pinpoint conservative active site residues which may contribute to divalent ion coordination and substrate binding.

## Introduction

*Helicobacter pylori* is one of the most widespread bacterial pathogens of humans, which infects approximately 50% of the world's population. *H. pylori* infection induces chronic gastric inflammation progressing to a variety of diseases ranging in severity from mild gastritis to peptic ulcers and some forms of gastric cancer [1], [2].

The complex pathology for various clinical outcomes has not been fully elucidated. It has been proposed that genetic variability may underlie the host adaptation differences of various *H. pylori* strains, which is reflected in distinct disease severities [3], [4]. Genome sequence comparisons in first fully sequenced *H. pylori* strains J99 and 26695 revealed plasticity zones in which nearly half the strain-specific genes of *H. pylori* are located [5]. With more complete genome sequence of *H. pylori* strains determined, the comparative analyses indicated that most strain-specific genes are preferentially localized to either plasticity regions or potential genome rearrangement sites [6]. Recently, there has been considerable interest in the strain-specific genes found in these plasticity regions. Studies have suggested that some genes are associated with the pathogenesis of *H. pylori* related diseases [7]–[9]. However, little is known about the function of many of the genes within the plasticity regions; thus, further studies are necessary to elucidate their roles in pathogenesis.

Many previous studies have focused on the plasticity region genes in *H. pylori* strain J99 (*jhp914–jhp961*) [10]. As studied, *jhp947* is significantly associated with duodenal ulcer and gastric cancer; therefore *jhp947* could be a good candidate marker for gastroduodenal diseases [7]. Another pathogenicity associated gene in the plasticity regions is *dupA (jhp917–jhp918)*, which encodes homologues of the VirB4 ATPase and is involved in both an increased risk for duodenal ulcer and reduced risk for gastric cancer [11]. Type IV secretion systems (T4SS) play important roles in DNA transfer contributing to bacterial genetic variability. *Tfs3* and *tfs4* have been successively identified and characterized as T4SS apparatus located in two different plasticity zones of *H. pylori* [6], [9], [12], [13].

*Jhp933* is one of the genes located in the plasticity region in J99 [14]. Analysis of *H. pylori* strains including strains 26695, J99 and HPAG1, *jhp933* has a prevalence rate of 51% [12]. The examination of plasticity region open reading frames (ORFs) in a small sample of gastritis and peptic ulcer patients revealed that the *jhp933* gene was found with a prevalence rate of 23.8% (5 of 21 patients) and 28.6% (4 of 14 patients), respectively [15].

The molecular details regarding the function of JHP933 are unknown due to the lack of sequence similarity with other well-characterized proteins. A BLAST search revealed that this protein is well conserved in some *Helicobacter* [Table S1 and Fig. S1] and closely related species. A conserved domain search indicated that JHP933 might be classified into the nucleotidyltransferase (NTase) superfamily, which constitutes a highly diverse superfamily of proteins with various important biological functions; including chromatin remodeling, RNA polyadenylation, RNA editing, DNA repairing, protein activity regulation, and antibiotic resistance [16]–[18]. Therefore, the specific biological function of JHP933 remains to be elucidated.

Here we have determined the crystal structure of JHP933 and revealed that JHP933 possesses a characteristic nucleotidyltransferase superfamily protein fold with a distinct, but conserved, active site. This structural description should contribute significantly to further uncovering the role of JHP933 in *H. pylori* pathogenesis.

## Materials and Methods

### Protein expression, purification, and crystallization

The gene encoding the full-length JHP933 from *Helicobacter pylori* strain J99 (NP_223650, 267 amino acids) was cloned into the modified pET15b vector (Novagen) and over-expressed as selenomethionyl protein in the *E. coli* strain BL21(DE3) using methionine pathway inhibition at 293 K. Bacterial cells were lysed by ultrasonication on ice in a buffer containing 50 mM Tris (pH 8.0), 300 mM NaCl, 5 mM β-mercaptoethanol, 0.1% Triton-X100 and 5% glycerol. Soluble N-terminally decahistidine-tagged JHP933 was bound to nickel-sepharose affinity resin. The eluted protein was further purified with size exclusion chromatography at 25 mM Tris (pH 8.0), 200 mM NaCl, 5 mM β-mercaptoethanol, 5% glycerol. The N-terminal histidine tag was removed by cleavage with TEV protease. Purified JHP933 was concentrated to 12 mg/mL without buffer exchange. SDS polyacrylamide gel electrophoresis of purified protein showed one major band at an approximate molecular weight of about 31 kDa, indicating pure full-length protein. Crystals were obtained with the sitting drop vapour-diffusion method at 293 K with 2 µL of protein mixed with 2 µL of a mother liquid solution containing 32% PEG4K, 0.1 M Potassium Sodium tartrate at 0.1 M HEPES (pH 7.5) buffer. Crystals were flash-frozen in liquid nitrogen with a mother liquid containing 25% PEG400 as cryoprotectant.

### Data collection, structure determination and refinement

The selenomethionyl single wavelength anomalous dispersion (SAD) dataset for JHP933 were collected at a wavelength of 0.9792 Å at 100 K on the BL17U1 beamline of the Shanghai Synchrotron Radiation Facility (SSRF) to a diffraction limit of 2.1 Å. Diffraction images and the anomalous data set were processed and scaled with HKL2000 [19]. SAD data processing statistics are summarized in Table 1. The locations of 6 selenium atoms were determined and an initial model built using the AutoSolve program of the Phenix suite [20]. The model was manually rebuilt with Coot [21] and further refined in Phenix. The final model contains residues 11–243, with refinement statistics summarized in Table 1. The Ramachandran statistics were calculated with Procheck [22]. Structure superimpositions were complemented by CCP4 LSQ superposion [23]. Figures were produced with Pymol (www.pymol.org). Multiple sequence alignments were generated manually or by using ESPript [24].

**Table 1:** Data collection and refinement statistics.

Data collection	SeMet
Space group	P 6_2_
Cell parameters	
a, b, c (Å)	90.06, 90.06, 70.87
α, β, γ (°)	90, 90, 120
Resolution (Å)	2.1
R_merge_ (%)	0.143 (0.756)
I/ơI	18.04 (2.40)
Completeness (%)	99.7 (100)
Redundancy	7.5 (7.4)
Wilson B-factor (Å^2^)	36.33
**Refinement**	
Resolution (Å)	2.100–38.005
No. reflections	18941 (1829)
R_work_/R_free_ (%)	20.43/23.08
No. atoms	
Protein	1867
Water	76
R.m.s.d bonds (Å)	0.008
R.m.s.d angles (°)	1.137
Ramachandran plot	
Favored (%)	96.96
Allowed (%)	3.04
Outliers (%)	0.00
Rotamer outliers (%)	0.00

Numbers in parentheses refer to the highest-resolution shell.

#### Accession Numbers

Coordinates and structure factors have been deposited in the Protein Data Bank with accession number 4O8S.

## Results and Discussion

The gene encoding the full-length JHP933 from *H. pylori* strain J99 was subcloned from genomic DNA, and the recombinant protein expressed as selenomethionyl protein in *E. coli* and purified using standard methods. Diffracting protein crystals were obtained and SAD diffraction data was used to solve the structure in the space group P62. The final crystal structure of JHP933, containing residues 11–243, was refined at a resolution of 2.1 Å with a R_work_ and R_free_ of 20.43% and 23.08%, respectively.

**Figure 1:**
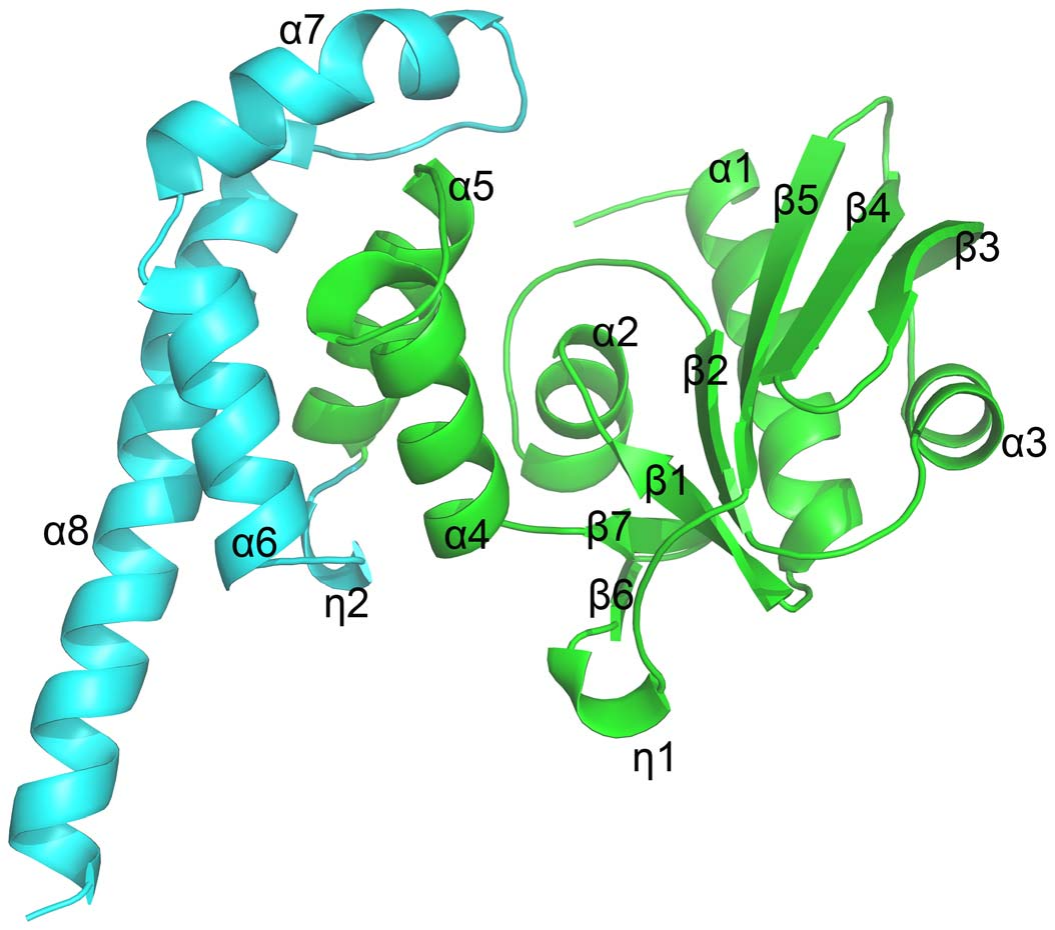
Overall structure of JHP933. Ribbon diagram of the JHP933 structure, N-terminal core domain is colored in lime and C-terminal tail domain in cyan. α-helices are labelled with α, β-strands are labelled with β, and 3_10_ helices are labelled with η.

The overall structure of JHP933 consists of two domains: an N-terminal core domain and a C-terminal tail domain [Fig. 1]. The N-terminal core domain covers residues 11–170 and contains 5 α-helices (α1–α5) and 7 β-strands (β1–β7). A 3_10_ helix (η1) connects β-strands β5 and β6. The C-terminal tail domain is formed by α-helices α6–α7 followed by an extended α-helix (α8). The two domains are connected by another 3_10_ helix (η2) between α-helices α5 and α6.

The N-terminal core domain of JHP933 has an αβαβαβα topology formed by α1-β1-α2-β2-α3-β5-α4, which is coincident with the common α/β-fold structure of nucleotidyltansferase (NTase) fold proteins (Fig. S2) [16]. For most NTase fold proteins, the core structure is usually decorated with various additional structural elements. In the JHP933 structure, the N-terminal core domain contains a seven-stranded, mixed β-sheet flanked by 4 α-helices, β1 and β2 forming antiparallel β-sheet, β2 and β5 forming parallel β-sheet, β5 forming antiparallel β-sheet with additional β-strands β3 and β4, and a stranded small β-sheet β6–β7 making a big turn linked to α-helices α4–α5 [Fig. 1 and S2].

**Figure 2:**
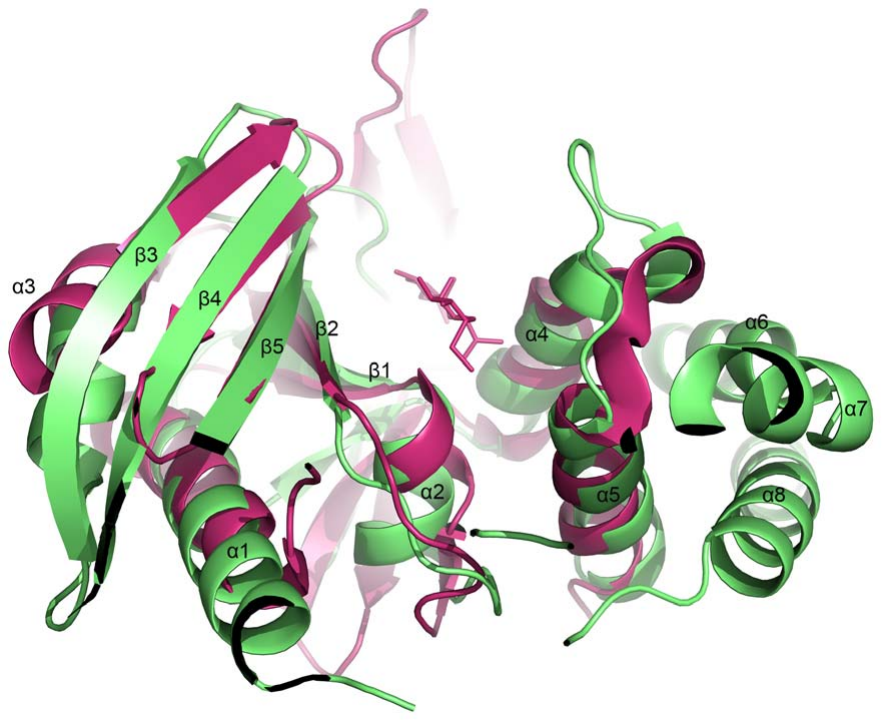
The superposition of JHP933 and LinA/Lincomycin complex (4E8J) structures. Ribbon diagram of JHP933/LinA, with JHP933 is colored in lime and LinA in magenta, and substrate lincomycin of LinA is shown in ball-and-stick representation.

**Figure 3:**
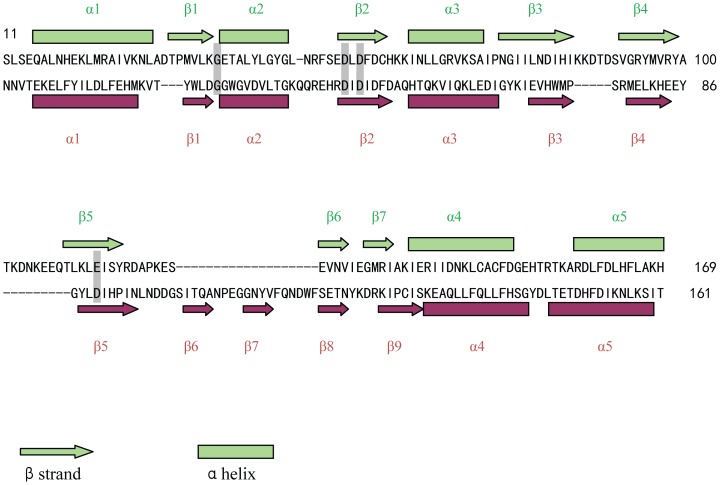
Sequence and secondary structure comparison of JHP933 with structurally related LinA. The secondary structures of JHP93 (top row) are labeled in lime and LinA from *S. haemolyticu* (bottom row) in magenta. The conserved active site motifs involved in catalysis ([DE]h[DE]h, h[DE]h) and substrate binding (hG) of NTase superfamily are shadowed in gray.

**Figure 4:**
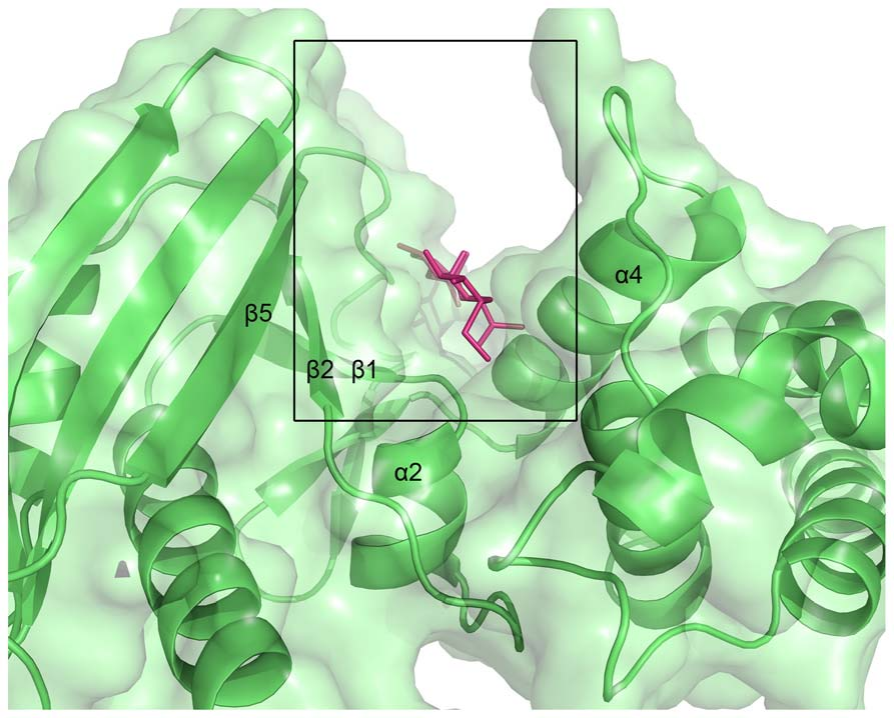
Putative substrate binding site of JHP933. Ribbon diagram and surface representation of JHP933 are colored in lime, the modelled substrate lincomycin of the superimposed LinA/lincomycin complex is shown in ball-and-stick representation and colored in magenta (LinA protein not shown).

A Dali search for structural homology identified lincosamide antibiotic adenylyltransferase LinA as the closest related structure with a Z-score of 9.6. LinA (pdb code: 4E8J) shares 16% sequence identity with JHP933 and superimposes with a Cα root-mean-square deviation (rmsd) of 2.7 Å over the N-terminal domain [Fig. 2 and 3]. The superposition of these two structures demonstrates a surprisingly high overall homology of the core structural elements including β-stands β1–β5 and the flanking α-helices α1–α4 in the N-terminal domain. The structural homology is highest in the core structure while significant differences can be seen in the addition of accessory structural elements and the loops which connect core elements [Fig. 2]. By comparison to the active site of LinA complex structure, a conservative large cleft is identified as a possible active site for substrate binding at the N-terminal core domain of JHP933. This substrate-binding cleft is surrounded mainly by β-strands β1, β2, β5 and α-helices α4, α2 with a size of around 13×20×20 Å [Fig. 4]. As LinA is a member of NTase superfamily, this high structural similarity further indicates that JHP933 should belong to the same superfamily.

**Figure 5:**
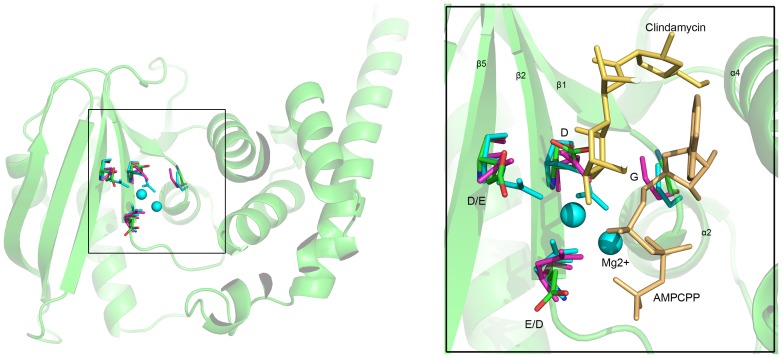
Active site conservation and substrate binding of JHP933, LinA and LinB. The C atoms of active site residues are shown in ball-and-stick representation and distinctively colored: lime for JHP933, magenta for LinA (4E8J), and cyan for LinB (3JZ0). The substrate Mg^2+^ ions, as cyan spheres, AMPCPP and clindamycin, in yellow, are from LinB complex structure.

Through sequence analyses of distinct members of NTase superfamily, a common sequence motif of active site residues has been noted: h[G/S], [D/E]h[D/E]h and h[D/E]h (h indicates a hydrophobic amino acid) [17]. The corresponding residues are G39, D55hD57 and E113 in JHP933; with G39 at the connection of β1 and α2, D55 and D57 located on β2, and E113 is placed on β5 structurally adjacent to β2 [Fig. 5]. To further clarify the active site and molecular mechanism for JHP933 substrate binding, we compared the structure of JHP933 N-terminal fragment with the LinA/lincomycin complex, in addition to another NTase fold protein LinB complexed with Mg^2+^, AMPCPP and clindamycin (pdb code: 3JZ0) [Fig. S3] [25]. The structural superpositions reveal that not only is the fold conserved but also position of catalytic residues. According to the superimposed structure of JHP933, the sites of G39, D55/D57 and E113 are strictly conserved 3-dimensionally [Fig. 5]. Conservation of the catalytic residues likely indicates a similar mechanism of action. Therefore, with reference to the structural conservation of these NTase superfamily proteins, the conserved G39 should play a crucial role in binding of substrates, and D55/D57 and E113 likely are involved in the coordination of divalent ions such as Mg^2+^, which chelates the phosphates of a nucleoside triphosphate substrate and plays a crucial role in activation of the second substrate's hydroxyl group [16], [25]. However, residues responsible for second substrate binding of LinB or LinA are not conserved in JHP933, likely reflecting differences in identity and structure of second substrates. However, the overall structure clearly confirms that JHP933 belongs to the NTase superfamily with the characteristic structural features well maintained.

In summary, the crystal structure of JHP933 of *H. pylori* strain J99 described here presents precise evidence to confirm JHP933 as a member of the nucleotidyltransferase superfamily. The structural information demonstrates that JHP933 conserves the overall fold of NTase superfamily proteins with an αβαβαβα topology and catalytic residues for substrate binding within conservative active site. Interestingly, from this large superfamily, we can observe that the proteins take a common core conformation though they display little sequence similarity and play diverse physiological roles.

Most NTase fold proteins can transfer nucleoside monophosphate (NMP) from substrate nucleoside triphosphate (NTP) to the hydroxyl group of their second substrate, such as a small molecule, nucleic acid or protein [17]. It is also worth noting that the overall fold of JHP933 and LinA is highly similar, which leads us to consider a role for JHP933 in lincosamide antibiotic resistance. A study of primary clindamycin resistance reported a prevalence rate of 13.1% in *H. pylori* strains from dyspeptic patients [26]. To date, mutations in the 23S rRNA gene are a clinically reported mechanism of resistance to lincosamide antibiotics in *H. pylori* [27]. However, for many bacteria, producing enzymes to modify antibiotics is a common mechanism of resistance for a number of classes of antibiotics. Given its structural similarity to LinA, it is possible that JHP933 may represent a, yet unobserved, mechanism of resistance; using the nucleotidyl transfer to modify antibiotics and inhibit their activity. However, this hypothesis needs further investigation as the putative substrate for JHP933 remains unknown. For a thorough understanding of JHP933's role in pathogenesis of *H. pylori* related diseases, this structural model represents a critical step in the description of JHP933 function.

## Supporting Information

Figure S1 A sequence alignment of JHP933 from strain J99 and the 20 closest orthologs (corresponding accession number see Table S1) found in other *H. pylori*.
(TIF)

Figure S2 Comparison of secondary structures of JHP933 and other nucleotidyltransferase fold proteins. JHP933 structure (top row) noting secondary structure elements and additional domains aligned with some representative NTase fold proteins of known structure (inside the frame and marked with pdb code, UniProtKB ID, and source organism). JHP933's secondary structure elements and the positions of conserved active site motifs involved in substrate binding (hG) and catalysis ([DE]h[DE]h, h[DE]h) are marked.
(TIF)

Figure S3 The sequence alignment for NTase superfamily core fragment of JHP933, LinA (UniProtKB ID: P06107, from *S. haemolyticu*) and LinB (UniProtKB ID: Q9WVY4, from *Enterococcus faecium*) from the top row to the bottom row. The secondary structural elements of JHP933 are illustrated.
(TIF)

Table S1 A BLAST search of JHP933 (marked with accession number) in fully sequenced *H. pylori* genomes.
(DOCX)
