# Supplementary figures and images for: Crystal Structure Confirmation of JHP933 as a Nucleotidyltransferase Superfamily Protein from Helicobacter pylori Strain J99
Source: PLoS One. 2014 Aug 7;9(8):e104609. doi: 10.1371/journal.pone.0104609 (PMC4125220; doi:10.1371/journal.pone.0104609)

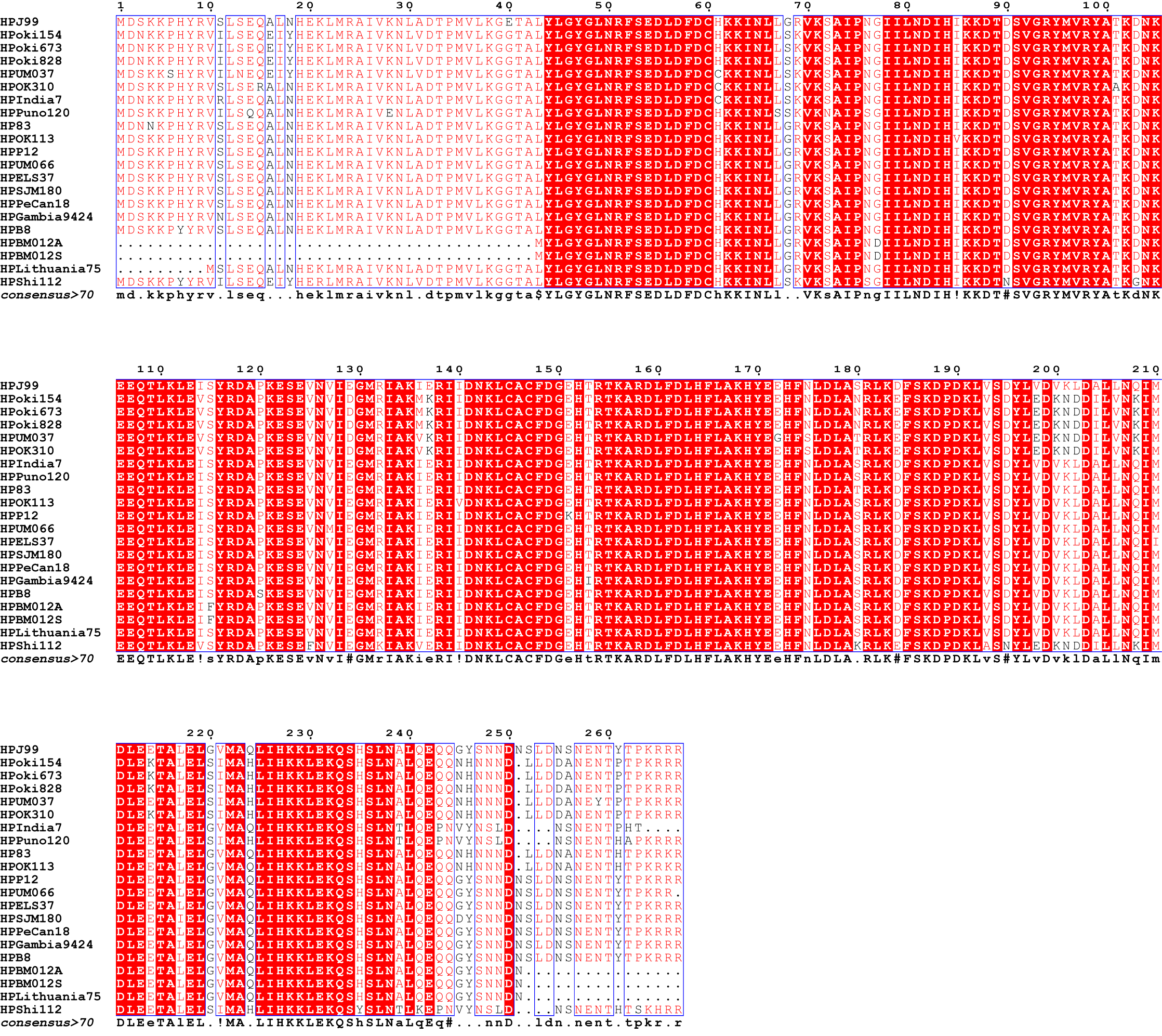

Supplement: Figure S1 — A sequence alignment of JHP933 from strain J99 and the 20 closest orthologs (corresponding accession number see Table S1) found in other H. pylori. (TIF) [file pone.0104609.s001.tif]

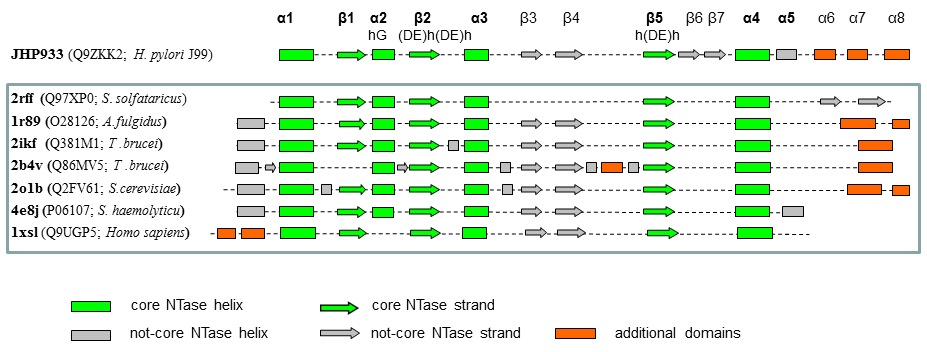

Supplement: Figure S2 — Comparison of secondary structures of JHP933 and other nucleotidyltransferase fold proteins. JHP933 structure (top row) noting secondary structure elements and additional domains aligned with some representative NTase fold proteins of known structure (inside the frame and marked with pdb code, UniProtKB ID, and source organism). JHP933's secondary structure elements and the positions of conserved active site motifs involved in substrate binding (hG) and catalysis ([DE]h[DE]h, h[DE]h) are marked. (TIF) [file pone.0104609.s002.tif]

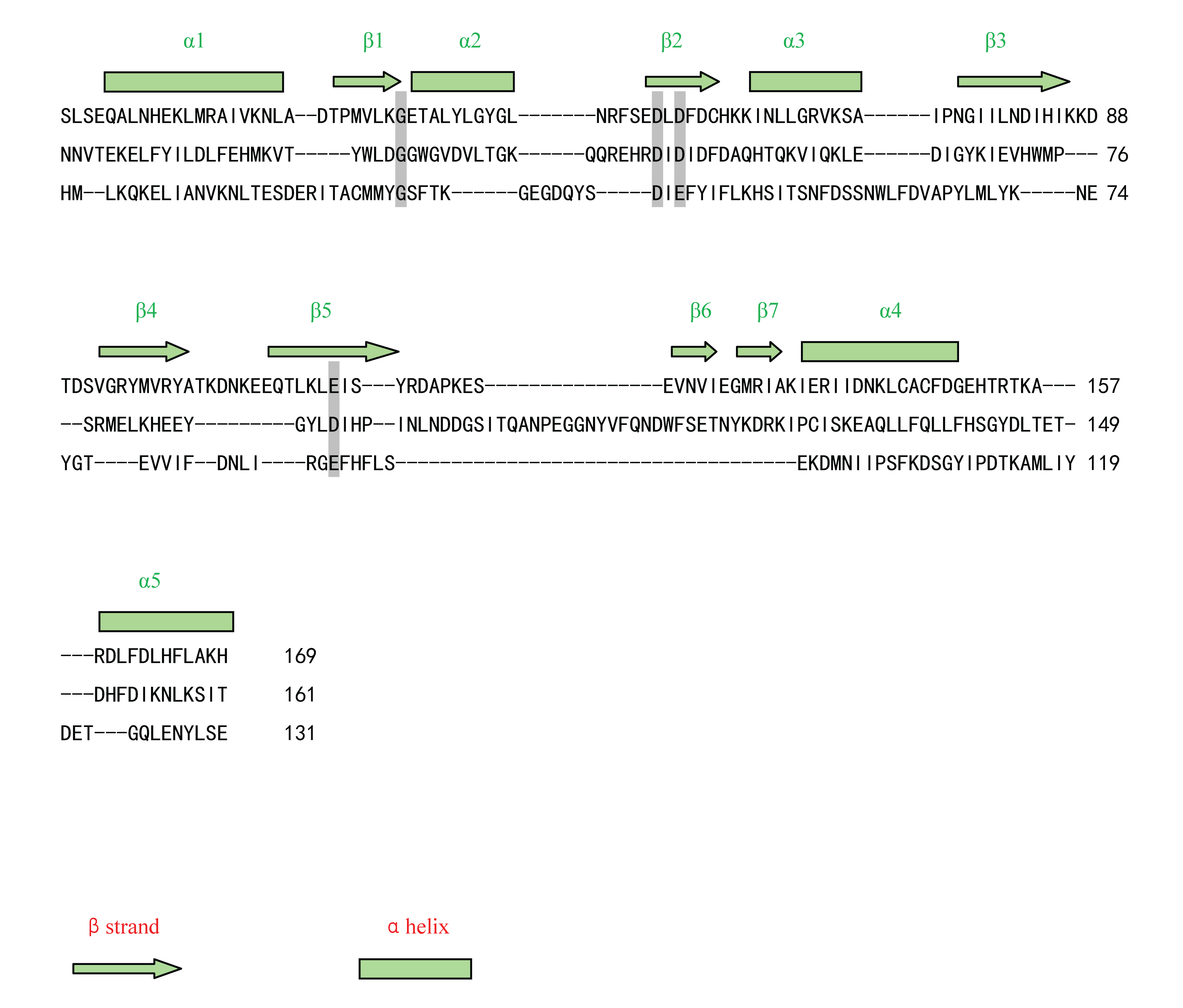

Supplement: Figure S3 — The sequence alignment for NTase superfamily core fragment of JHP933, LinA (UniProtKB ID: P06107, from S. haemolyticu) and LinB (UniProtKB ID: Q9WVY4, from Enterococcus faecium) from the top row to the bottom row. The secondary structural elements of JHP933 are illustrated. (TIF) [file pone.0104609.s003.tif]
