# Supplementary material for: Crystal Structure Confirmation of JHP933 as a Nucleotidyltransferase Superfamily Protein from Helicobacter pylori Strain J99
Source: PLoS One. 2014 Aug 7;9(8):e104609. doi: 10.1371/journal.pone.0104609 (PMC4125220; doi:10.1371/journal.pone.0104609)
Supplement: Table S1 — A BLAST search of JHP933 (marked with accession number) in fully sequenced H. pylori genomes. (DOCX) [file pone.0104609.s004.docx]

Supplementary Table S1. A BLAST search of JHP933 (marked with accession number) in fully sequenced *H. pylori* genomes.

| **Strain** | **Accession** | **Coverage**  **(%)** | **Identity**  **(%)** | **Strain** | **Accession** | **Coverage**  **(%)** | **Identity**  **(%)** |
| --- | --- | --- | --- | --- | --- | --- | --- |
| HPJ99 | NP_223650 | 100 | 100 | HP Cuz20 | YP_005767491 | 9 | 48 |
| HP B8 | YP_003728556 | 100 | 99 | HP Sat464 | YP_005768982 | 9 | 48 |
| HPEls37 | YP_005424961 | 100 | 99 | HP Xz274 | YP_006338205 | 7 | 48 |
| HP Gambia94/24 | YP_005781139 | 100 | 99 | HP V225d | YP_005764353 | 9 | 48 |
| HP UM066 | YP_007984590 | 100 | 99 | HP Oki128 | AHN37676 | 7 | 48 |
| HP P12 | YP_002301970 | 100 | 99 | HPUM032 | YP_007978261 | 7 | 48 |
| HP Pecan18 | YP_006229216 | 100 | 99 | HP 52 | YP_005762648 | 9 | 46 |
| HP SJM180 | YP_003929263 | 100 | 99 | HP 908 | YP_005765743 | 9 | 46 |
| HP Lithuania75 | YP_005773647 | 90 | 99 | HP F16 | YP_005779417 | 9 | 46 |
| HP BM012A | YP_008826720 | 78 | 99 | HP F30 | YP_005773894 | 9 | 46 |
| HP BM012S | YP_008846049 | 78 | 99 | HP F57 | YP_005777921 | 9 | 46 |
| HP 83 | YP_005784660 | 100 | 95 | HP G27 | YP_002266809 | 9 | 46 |
| HP F32 | YP_005776225 | 100 | 95 | HP HPAG1 | YP_627932 | 9 | 46 |
| HP india7 | YP_005782073 | 97 | 95 | HP Pecan4 | YP_003927486 | 9 | 46 |
| HP Puno120 | YP_005789146 | 100 | 92 | HP Rif1 | YP_006893734 | 9 | 46 |
| HP Ok310 | YP_007539022 | 100 | 88 | HP Rif2 | YP_006936775 | 9 | 46 |
| HP Oki154 | AHN38832 | 100 | 87 | HP Shi169 | YP_006226398 | 9 | 46 |
| HP Oki673 | AHN41737 | 100 | 87 | HP Shi470 | YP_001910725 | 9 | 46 |
| HP Oki828 | AHN43175 | 100 | 87 | HP SouthAfrica7 | YP_005770845 | 9 | 46 |
| HP UM037 | YP_007981487 | 100 | 85 | HP UM298 | YP_008337074 | 9 | 46 |
| HP Shi112 | YP_006226985 | 100 | 84 | HP UM299 | YP_007981262 | 9 | 46 |
| HP Hup-B14 | YP_006220010 | 8 | 68 | HP Oki102 | AHN35201 | 9 | 46 |
| HP Aklavik117 | YP_007016986 | 5 | 67 | HP Oki898 | AHN45350 | 9 | 6 |
| HP Shi417 | YP_006225110 | 7 | 58 | HPSouthAfrica20 | YP_008471290 | 9 | 44 |
| HP B38 | YP_003057326 | 7 | 52 | HP SNT49 | YP_005787008 | 12 | 44 |
| HP Puno135 | YP_005790627 | 9 | 52 | HP Oki112 | AHN36013 | 12 | 44 |
| HP 2017 | YP_005783604 | 8 | 50 | HP 35A | YP_005769371 | 10 | 43 |
| HP2018 | YP_005791448 | 8 | 50 | HP Oki422 | AHN40109 | 7 | 43 |
| HP 51 | YP_005792919 | 7 | 48 | HP 26695 |  |  | 0 |
| HP Aklavik86 | YP_007018318 | 9 | 48 | HP B45 |  |  | 0 |
